# Imbalanced Frequencies of Th17 and Treg Cells in Acute Coronary Syndromes Are Mediated by IL-6-STAT3 Signaling

**DOI:** 10.1371/journal.pone.0072804

**Published:** 2013-08-26

**Authors:** Yanhui Ma, Xiangliang Yuan, Lin Deng, Weiping Xu, Yingxia Zheng, Chaoyan Yue, Guanghui Zhang, Fang Xie, Yuan H. Yang, Michael P. Gantier, JunPing Liu, Dakang Xu, Lisong Shen

**Affiliations:** 1 Department of Clinical Laboratory, Xinhua Hospital, Shanghai Jiao Tong University School of Medicine, Shanghai, China; 2 Department of Internal Cardiology, Xinhua Hospital, Shanghai Jiao Tong University School of Medicine, Shanghai, China; 3 Centre for Inflammatory Diseases, Department of Medicine, Monash University, Melbourne, Australia; 4 Monash Institute of Medical Research, Monash University, Melbourne, Australia; 5 Institute of Ageing Research, Hangzhou Normal University School of Medicine, Hangzhou, China; Université de Montréal, Canada

## Abstract

**Aims:**

Extensive evidence suggests inflammatory components participate in the pathogenic processes of acute coronary syndromes (ACS). In this study, we aimed to elucidate the role and mechanism underlying the imbalance of Th17 and Treg cell peripheral populations in the pathogenesis of ACS.

**Methods and Results:**

Using a flow cytometric analysis, we observed a significantly increased frequency of Th17 cells and a concurrently decreased CD4^+^CD25^+^Foxp3^+^ Treg cells in patients with ACS. To elucidate the mechanism of Th17/Treg imbalance in ACS, 22 inflammatory cytokines were measured using multiplexed immunobead-based assays. Of six elevated cytokines in ACS patients, only IL-6 was positively correlated with a higher Th17 cell level (*r* = 0.39, *P*<0.01). Relying on IL-6 stimulating and neutralizing studies, we demonstrated a direct role for IL-6 in sera from ACS patients with an increased frequency of Th17 cells. IL-6 induces the differentiation of Th17 cells from naïve CD4^+^ T cells through STAT3 activation and RORγt induction. However, we observed that high levels of TGF-β1 inhibited IL-6-dependent Th17 cell differentiation, indicating a complex interplay between the two cytokines in the control of Th17 and Treg cell populations.

**Conclusions:**

Our results demonstrate the role of IL-6-STAT3 signaling in ACS through increased Th17 cell differentiation. These findings indicate that IL-6 neutralizing strategies could present novel therapeutic avenues in the treatment of ACS.

## Introduction

Extensive evidence supports a pathogenic role for both local and systemic inflammation in the origin and progression of atherosclerosis and in the pathogenesis of acute coronary syndromes (ACS) [1]. Complex interplays between Th1 and Th2 lymphocytes are known to contribute to the pathophysiology of ACS. In addition to Th1 and Th2 cells, there is accumulating evidence for a critical role for Th17 and Treg cell subsets in the regulation of the immune system [2]. The pro-inflammatory cytokine IL-17, which is produced by Th17 cells and other innate immune cells, has been implicated in many inflammatory conditions [3]. Conversely, Treg cells repress inflammation through the modulation of T-cell-mediated responses. Previous studies have established that cells expressing both IL-17 and IFN-γ are present in patients with ACS [4]. It has also been proposed that increased Th17 cell levels correlate with decreased Treg cell levels, suggesting that the imbalance between these two subsets may contribute to the pathogenesis of atherosclerosis in humans [5]. Nevertheless, the effects of such variation in the ratio of Th17/Treg cells and the mechanisms underlying its imbalance effects in ACS remain poorly understood.

Among circulating CD4^+^ T lymphocytes, we and others have observed that natural Treg populations can be distinguished from activated CD4^+^CD25^+^ T cells on the basis of their CD127^low^FOXP3^+^ phenotype [6]–[8]. It was shown that Th17 cells counteract the effect of natural Treg cells in the maintenance of self-tolerance [9]. Differentiation of Tregs and Th17 cells relies on specific transcription factors executing Treg or Th17 programs, such as FOXP3 and STAT3/RORγt, respectively. Although functionally distinct, several studies have now established the existence of reciprocal developmental pathways for Th17 and Treg cells. In humans, Th17 differentiation from naïve CD4^+^CD25^−^ T cells was originally shown to be induced by a combination of IL-1β and IL-6 but was shown to be suppressed by TGF-β1 *in vitro* [10]–[11]. In contrast, the association of IL-6 and TGF-β1 promoted differentiation of Th17 cells in mice, whereas TGF-β1 alone promoted differentiation of Treg cells [12]–[13], suggesting a pivotal role for TGF-β1 in the differentiation of naïve CD4^+^CD25^−^ T cells into Th17 or Treg cells. Recent reports have shown that combinations of TGF-β1 with IL-21 or IL-1β with IL-23 can induce the differentiation of Th17 cells from human naïve CD4^+^ T cells [14]–[16]. As shown with the effects of IL-1β and IL-6 previously mentioned, monocyte-derived pro-inflammatory cytokines have been shown to directly contribute to the differentiation of conventional CD4^+^ T cells into Th17 cells [17]–[18]. In addition, other pro-inflammatory cytokines, such as IL-12, can suppress the differentiation of Th17 cells. Nevertheless, the precise roles of the differentiation factors (TGF-β plus IL-6 or IL-21), the growth and stabilization factor (IL-23) and other cytokines in the differentiation and function of Th17 cells are not yet fully understood. The relationship between Th17 and Treg cells, however, is complex and has not been completely elucidated. In particular, how the interplay between the two subsets influences chronic inflammation is of great interest in diseases such as ACS.

Here, we investigate the mechanisms underlying the imbalance of Th17 and Treg cell populations in patients with ACS. In particular, we assessed the role of pro-inflammatory cytokines in the establishment of the imbalance between Th17 and Treg cell populations and explored the detailed signaling pathway for the differentiation of Th17 cells in ACS patients. We uncovered a critical role for IL-6 signaling in the positive regulation of Th17 cell differentiation. These findings identify a novel role for IL-6 in the pathogenesis of ACS.

## Materials and Methods

### Patients and controls

A total of 67 patients with coronary artery disease (CAD), who were diagnosed by coronary angiography and displayed one or more coronary arteries with at least 50% stenosis, were enrolled in this study in the Department of Internal Cardiology and Emergency from Xinhua Hospital, Shanghai Jiaotong University School of Medicine. Patients were classified into ACS (n = 51) and stable angina (SA) (n = 16) subgroups. ACS was defined as a spectrum of CAD that included ST-elevation myocardial infarction (STEMI), non-ST-elevation myocardial infarction or unstable angina pectoris (NSTEMI/UA) with typical symptoms. Diagnoses were made based on clinical presentation, presence of electrocardiogram (ECG) changes and testing positive for cardiac troponins, according to the guidelines of the European Society of Cardiology (ESC) for the management of STEMI and NSTEMI/UA [19]. Diagnosis of SA was defined by typical effort angina symptoms associated with downsloping or a horizontal ST-segment depression greater than 1 mm in an exercise test. The Gensini score was calculated to evaluate severity of coronary artery disease [20]. Twenty-five age- and sex-matched healthy donors (HD) who served as controls were obtained from the medical examination center of Xinhua Hospital. The following exclusion criteria were used: myocardial bridging, renal failure, serum creatinine above 2 mg/dL, known history of cancer, chronic immune-mediated disorders or current use of immunosuppressive agents, including corticosteroids. The demographic and risk factor information of all case and control samples are summarized in Table 1. For further analyses, we sub-divided the cohort of ACS patients into three groups according to their levels of circulating IL-6 and TGF-β1. This study was approved by the Ethics Committee of Xinhua Hospital, Shanghai Jiao Tong University School of Medicine, and written informed consent was obtained from all patients prior to their participation. All studies were performed in accordance with the Declaration of Helsinki.

**Table 1:** Clinical characteristics of the groups.

Characteristics	HD (n = 25)	SA (n = 16)	ACS (n = 51)
Age, mean ± SD years	56.5±13.9	65.9±8.1	67.4±11.3
Gender (Male/Female)	17/8	10/6	39/12
Gensini score, mean ± SD	N/A	15.1±10.5	44.2±26.7^a^**
Vessel lesions (1/2/3 vessels, n)	N/A	8/5/3	19/22/10
Risk factors, n(%)			
Hypertension	N/A	9(56.25%)	35(68.63%)
Diabetes mellitus	N/A	5(31.25%)	12(23.53%)
Current smoker	N/A	4(25%)	22(43.14%)
Drinking history	N/A	1(6.25%)	10(19.61%)
Biochemical cardiac markers,			
median (Q1, Q3)			
TNI (ng/ml)	0.012(0.009, 0.020)	0.01(0.006, 0.020)	0.03(0.009, 0.881)
Hs-CRP (ng/ml)	0.32(0.08, 0.63)	1.19(0.46, 3.51)*	2.59(0.51, 8.79)**
LDL (mmol/L)	2.61(2.15, 3.07)	2.43(2.09, 2.96)	2.62(2.34, 2.88)
Lp(a) (mg/dL)	3.90(1.75, 11.85)	4.75(1.11, 21.43)	8.20(4.90, 19.00)*

Except where indicated otherwise, values are the number (%). NA = not applicable. * *P*<0.05; ** *P*<0.01 was statistically significant by a two-tailed test, ^a^ compared with the SA or otherwise compared to HD.

### Blood sampling and measurements

The blood samples used for this study were collected at the time of admission to the Emergency Department or coronary care unit (CCU) of the Internal Cardiology Department (<24 h from symptom onset) before coronary angiography. Sera derived from patients and HDs for cytokine measurements were collected and frozen at −80°C until used. Clinical biomarkers were immediately measured in the clinical chemistry laboratory of Xinhua Hospital, including differential white cell count, low-density lipoprotein (LDL), cholesterol (CHO), high-sensitivity CRP (hs-CRP), lipoprotein(a) (Lp(a)), and glucose. Troponin I (TNI) was measured by an enhanced TNI assay (Beckman Coulter, CA, USA). The serum levels of IL-1α, IL-1β, IL-2, IL-3, IL-4, IL-5, IL-6, IL-8, IL-10, IL-12(40), IL-12(70), IL-13, IL-15, IL-17, IFN-γ, IFN-α, tumor necrosis factor-α (TNF-α), and TNF-β were measured using immunobead-based 16-plex assays (Luminex) with a detection limit at 2.5 pg/mL. TGF-β1 and IL-6 levels were re-detected quantitatively using enzyme-linked immunosorbent assay (ELISA) kits (R&D Systems), according to the manufacturer's instructions. The detection limit of IL-6 and TGF-β1 was 0.7 pg/ml and 15 pg/ml, respectively.

### Flow cytometric analysis

PBMCs (peripheral blood mononuclear cells) were freshly isolated by Ficoll density gradient centrifugation and re-suspended in PBS supplemented with 2% bovine serum albumin at a concentration of 1×10^6^ cells/ml. Fluorochrome-labeled mouse anti-human monoclonal antibodies targeted against CD3-PC7, CD4-PerCP and CD25-APC were purchased from Beckman Coulter. FITC- and PE-conjugated antibodies specific to IL-17, IFN-γ, retinoid-related orphan receptor C (RORγt), and pSTAT3 were purchased from BD. Foxp3-FITC and IgG2a-FITC (eBioscience, CA, USA) were used, together with appropriate isotype controls, to identify the positive and negative cell populations. For the intracellular staining of IL-17 and IFN-γ, cells were stimulated for 4 h with PMA (50 ng/ml) and ionomycin (1 µg/ml) (Sigma-Aldrich) in the presence of monensin (Golgi-Stop, BD). The cells were harvested, washed, and stained with anti-CD4 or anti-CD8 antibodies in the presence of FcR-Block (BD). After washing, the cells were fixed, permeabilized, and stained with cytokine-specific or control isotype antibodies for 30 min on ice. To analyze the levels of STAT3 under each of the four conditions in ACS patients, PBMCs were left unstimulated or were treated with IL-6 (100 ng/ml) as a positive control. For further studies, HD PBMCs were incubated with 20% ACS sera or were treated with an anti-IL-6 monoclonal antibody (mAb). Cells were fixed with 16% formaldehyde (final concentration of approximately 1.5%) for 30 min, permeabilized with 1 ml of ice-cold methanol for 30 min on ice, and stained with phosphospecific antibodies against STAT3 (pY705) (Figure S1 in File S1). A multiple-color flow cytometric analysis was performed using a BD FACS Aria flow cytometer (BD). For the analysis and visualization of cell density plots, the analysis software Cytobank was used (https://cytobank.stanford.edu). Normalized background-subtracted FCS files were imported into Cytobank for single-cell and population gating. Cytobank was also used to create heat maps comparing the fluorescence of the stimulated population by phospho-STAT3 mean fluorescence intensity (MFI) [21].

### Cell sorting and functional assays

For cell sorting, 10 ml of peripheral blood was collected from either HDs or ACS patients, and naïve T cells (CD4^+^CD45RA^+^CD45RO^−^) and memory T cells (CD4^+^CD45RO^+^CD45RA^−^) were obtained from PBMCs using the human Naive CD4^+^ T Cell Isolation Kit II and Memory CD4^+^ T Cell Isolation Kit (Miltenyi Biotec, Bergisch-Gladbach, Germany). The purity of the cells was 90% or greater as determined by re-analysis (Figure S2 in File S1). Typically, the cells were stimulated with precoated 5 µg/ml αCD3 and soluble 5 µg/ml αCD28 at 10^5^ per well in 96-well U-bottom plates, and three replicate wells were set up. The cells were cultured in a humidified CO_2_-containing atmosphere at 37°C for 7 days in quintuplicate with 20% ACS patient sera and complete RPMI-10 medium supplemented with 100 U/ml penicillin, 100 µg/ml streptomycin, 0.5 mM sodium pyruvate, 0.05 mM nonessential amino acids, 2 mM L-glutamine, and 10 mM HEPES (all from GIBCO). To analyze the factors responsible for the regulation of Th17 cell differentiation, rhIL-6 and TGF-β1 (R&D Systems) were added at a final concentration of 20 ng/ml and 10 ng/ml, respectively, as controls. For the IL-6 blockade experiment, neutralizing anti-IL-6 mAb (R&D Systems) was added at a concentration of 2 µg/ml. For the culture experiment, sera were pooled together from 8–12 individuals of each group.

### Real-time PCR

Total RNA was isolated with Qiagen reagents. Then, first-strand cDNA was subsequently synthesized using the Sensiscript RT Kit (TaKaRa), according to the manufacturer's instructions. Levels of mRNA for RORγt, Foxp3 and selected pro- or anti-inflammatory molecules were determined by real-time PCR with a SYBR Green master mix (TaKaRa). Data were collected and quantitatively analyzed, and the human GAPDH gene was used as an endogenous control for sample normalization. The sequences of each primer may be found in the SI (Table S1 in File S1).

### Statistical analysis

Continuous clinical data were expressed as the mean ± standard deviation (SD), and if there was a skewed distribution, the results were expressed as median and percentiles. All *in vitro* experiment data with five repeats were expressed as the mean ± SEM. Two-tailed Student's *t* test and an ANOVA were performed to analyze the differences between the groups. For normally distributed data, differences between groups were evaluated by Tukey's test, and association was assessed by Pearson's correlation coefficient. For non-normally distributed data, differences between groups were evaluated by the non-parametric Mann-Whitney U test, and association analysis was assessed using a Spearman rank correlation coefficient. A value of *p*<0.05 was considered statistically significant. All of these analyses were performed using GraphPad Prism (version 5.0).

## Results

### Elevated Th17 and decreased Treg cell levels in patients with ACS

**Figure 1:**
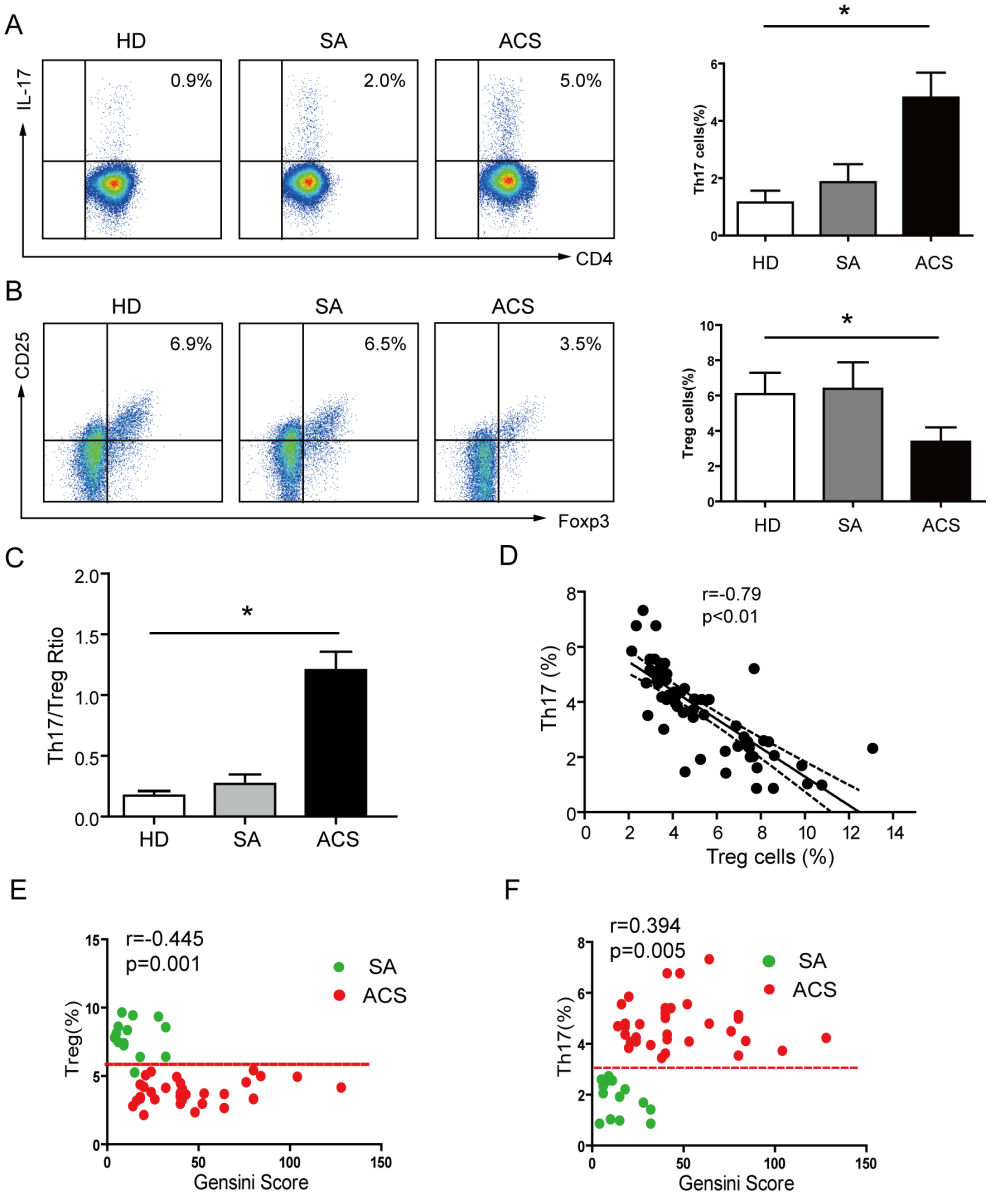
Imbalanced frequencies of Th17 and Treg cells in patients with ACS. *(A)* Representative flow cytometric (FCM) dot plots of intracellular IL-17 staining. IL-17 expression was determined by FCM gating of CD3^+^CD4^+^ cells. A summary of the percentages of CD4^+^IL-17^+^ T cells in different groups of patients with ACS is shown (HD, n = 25; SA, n = 16; ACS, n = 51). *(B)* Representative FCM dot plots of Treg cell quantification. Treg cells are defined as CD25^+^FOXP3^+^ double-positive cells. A summary of the percentages of Treg cells in different groups of patients with ACS is shown; *p*<0.05 compared with HDs. *(C)* The ratio of Th17 to Treg cells was significantly increased in patients with ACS. *(D)* Increased frequencies of Th17 cells in ACS patients were inversely correlated with the percentages of Treg cells. Scatter plots of Th17 frequencies and Treg frequencies with the Gensini Score. *(E)* A significant positive correlation between Th17 and the Gensini score was identified. *(F)* Treg cell frequencies negatively correlate with the Gensini score. Pearson's correlation coefficient (normal distributed data) and Spearman's rank correlation coefficient (non-normal data) were used to assess interrelationships. *: *P*<0.05 is considered statistically significant.

To determine the levels of Th17 and Treg cells in ACS patients, we analyzed the proportion of Th17 cells among total CD4^+^ T cells in PBMCs from ACS and HD controls [6], [22]. As shown in Figure 1A, the frequencies of Th17 cells were markedly higher in patients with ACS (4.81% ± 0.87%) compared with those with SA (1.89% ± 0.67%, *P*<0.05) and the HD control group (1.15% ± 0.38%, *P*<0.05) – both the SA and HD groups had comparable amounts of Th17 cells (*P*>0.05). In parallel, we analyzed the levels of Treg cells in CD4^+^ T cells, according to their level of CD25^+^FOXP3^+^ expression. As shown in Figure 1B, the proportion of CD25^+^FOXP3^+^ Tregs in the total PBMCs from healthy controls ranged from 3.37% to 9.23% (6.16% ± 1.33%), which was comparable with the SA group (6.38% ± 1.45%). However, there was a marked decrease of CD25^+^FOXP3^+^ Tregs in patients with ACS (3.34% ± 0.76%) (*P*<0.05). The relative proportions of Th17 to Treg cells were further compared for each group (Fig. 1C). Similar ratios of Th17/Treg cell populations were observed in both HD and SA groups, whereas higher frequencies of Th17 cells with lower frequencies of Tregs resulted in enhanced Th17/Treg ratios in the ACS groups (*P*<0.01). Accordingly, increased frequencies of Th17 cells negatively correlated with that of Treg cells in individual with ACS (r = −0.79, *P*<0.05) (Fig. 1D).

To investigate whether upregulated Th17 and deregulated Treg levels related to the severity of CAD, a bivariate correlation analysis was performed in ACS and SA that measured the Treg and Th17 percentage with a Gensini score [20], [23], which was associated with the severity of CAD. In particular, a significant positive linear relationship between Th17 and the Gensini score was demonstrated (*r* = 0.394, *P* = 0.0051) (Fig. 1E). The Treg level was inversely correlated with the Gensini score (*r* = −0.445, *P* = 0.0013) (Fig. 1F). Overall, these results confirm the existence of an imbalance between Treg and Th17 cells in patients with ACS, and these patients were chosen to participate in the following experiments.

### Elevated levels of IL-6 and TGF-β1 correlate with the imbalance of Treg and Th17 cell populations in ACS patients

**Figure 2:**
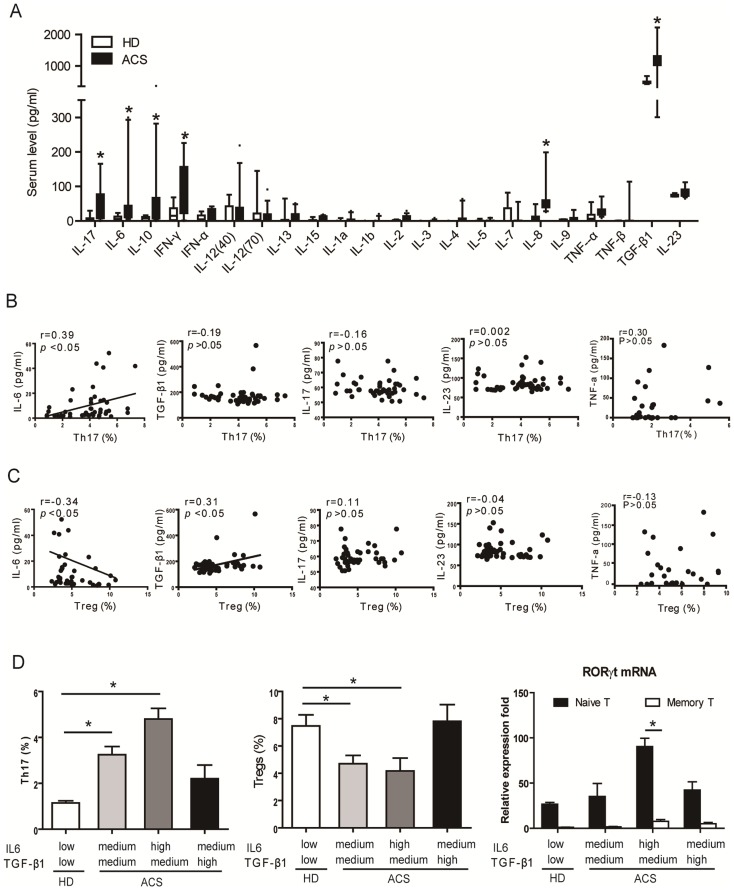
Pro-inflammatory cytokines regulate Treg and Th17 cell differentiation. *(A)* The levels of pro-inflammatory cytokines in the sera of healthy donors (n = 25) and ACS patients (n = 51) were determined by high-sensitivity multiplex assays. The results are shown as the median (10–90 percentiles). *(B)* Individual frequencies of Th17 cells positively correlate with circulating IL-6 levels in patients with ACS (n = 51). The TGF-β1, IL17 and IL23 levels were not associated with the frequency of Th17 cells. Correlations were determined by Spearman's rank correlation coefficients. The relationships are also depicted using linear regression (solid line). *(C)* Circulating IL-6 levels negatively correlate with the proportion of Treg cells. In addition, TGF-β1 concentrations positively correlate with the proportion of Treg cells. *(D)* Comparisons of the frequencies of Th17 and Treg cells in ACS patients (n = 51); RORγt mRNA expression in naive and memory T cells (n = 20) with different deliberately divided serum levels of IL-6 and TGF-β1. (IL-6: low, 0–10 pg/ml; medium, 10–50 pg/ml; high, >50 pg/ml; TGF-β1: low, 0–200 pg/ml; medium, 200–1000 pg/ml; high, >1000 pg/ml. IL-6 and TGF-β1 were determined by ELISA.). *: *P*<0.05 is considered statistically significant.

The cytokine environment during the development of ACS may favor the generation of pathological Th17 cells. To investigate this possibility, the levels of 22 cytokines, including IL-6, TGF-β1, IFN-γ, IL-12, IL-10, IL-1β, IL-17, IL-23, and others were measured in sera from patients with ACS (*n* = 51) and compared with sex- and age-matched HDs (*n* = 25) by high sensitivity multiplex arrays [24]. Levels of circulating pro-inflammatory cytokines, including IL-6, IFN-γ, IL-17 and IL-8, together with the levels of TGF-β1, were significantly increased in patients with ACS compared with HDs (Fig. 2A). However, no significant differences were observed in the levels IL-1β and IL-23, which have been reported to play critical roles in Th17 cell differentiation (Table 2). TGF-β1, IL-6, IL-17 and IL-23 are critical for the development and function of mouse Th17 cells [25]. We further characterized the putative role of these cytokines in the differentiation of Th17 cells in patients with ACS by establishing the correlation between individual levels of these cytokines and Th17 cell levels (Fig. 2B). Unlike all of the other cytokines measured, the circulating levels of IL-6 strongly correlated with the frequency of Th17 cells in ACS patients (Fig. 2B). Reciprocally, individual IL-6 levels inversely correlated with Treg cell numbers (Fig. 2C). In addition, circulating TGF-β1 levels positively correlated with numbers of Treg cells in ACS (Fig. 2C). In contrast, the levels of circulating IL-17 did not correlate with the numbers of Th17 or Treg cells (Fig. 2B&C). Therefore, these results indicate that IL-6 and TGF-β1 may have a role in the regulation of the imbalance between the Th17 and Treg cell populations observed in patients with ACS.

**Table 2:** The correlation of Th17 and Treg cells with serum cytokines in patients with ACS.

Cytokines	Th17 Cells		Treg Cells	
	r	*P*	r	*P*
IL-6	0.39	<0.01*	−0.34	0.03*
TGF-β1	−0.19	0.52	0.31	0.03*
IL-17	−0.16	0.50	0.11	0.57
IL-23	0.24	0.41	0.31	0.38
IFN-γ	0.20	0.51	−0.23	0.46
IL-10	−0.22	0.50	0.25	0.41

* *P*<0.05 represents a significant correlation using the Spearman method (r coefficients and probability values).

In further analyses, we sub-divided the cohort of ACS patients into three groups according to their levels of circulating IL-6 and TGF-β1 (Figure S3 in File S1). A comparison of the frequencies of Th17 and Treg cell populations in each group revealed that increased IL-6 levels were reflective of increased levels of Th17 cells, and reduced levels of Treg cells were consistent with RORγt mRNA expression, especially in naive T cells (Fig. 2D). Importantly, however, high levels of TGF-β1 inhibited the effects of IL-6 on Th17 cell populations in ACS patients. To understand the sources of IL-6 and TGF-β1, ACS patients were divided into three groups: UA, STEMI and NSTEMI. IL-6 was significantly higher in the UA, STEMI and NSTEMI groups than among the HD and SA groups, and there was more IL-6 in the STEMI and NSTEMI groups; however, there was no significant difference between these two groups (Table 3). TGF-β1 demonstrated no significant difference among these groups (Table 3).

**Table 3:** Concentrations of serum IL-6 and TGF-β1 in ACS patients and controls.

	IL-6 (pg/ml)	*P*	TGF-β1 (pg/ml)	*P*
	Median (Q1,Q3)		Median (Q1,Q3)	
HD (n = 25)	1.22 (0.41, 2.89)	/	163 (142, 187)	/
SA (n = 16)	3.44 (2.00, 5.33)	>0.05^a^	177 (162, 252)	>0.05^a^
UA (n = 18)	6.89 (2.67, 12.77)	<0.05^a^	138 (122, 312)	>0.05^a^
STEMI (n = 18)	54.78 (19.90, 98.49)	<0.01^a^	109 (185, 2821)	>0.05^a^
NSTEMI (n = 15)	25.11 (5.11, 43.89)	<0.01^a^; >0.05^b^	180 (150, 3209)	>0.05^a, b^

*P*<0.05, *P*<0.01 is statistically significant according to a two-tailed test ^a^ compared with HD ^b^ and compared to the STEMI group.

### IL-6 induces the differentiation of Th17 cells from naïve T cells through STAT3 activation and RORγt induction

**Figure 3:**
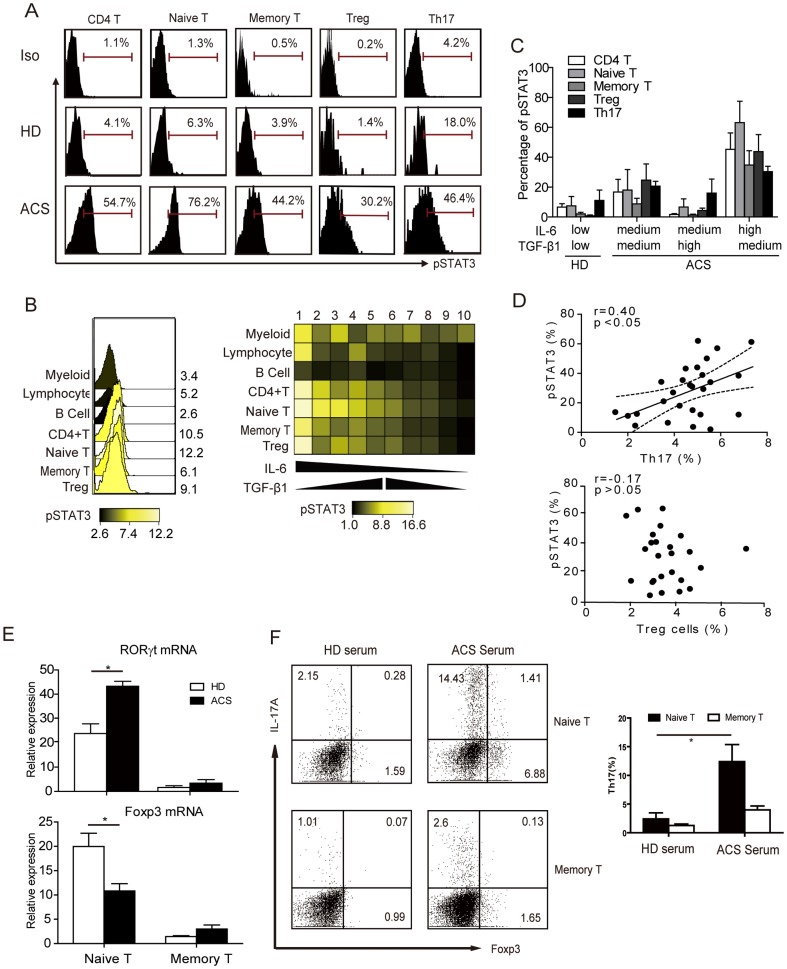
IL6-STAT3 signaling in patients with ACS. *(A)* Representative FCM histograms of pSTAT3 levels in CD4^+^ T cells, naïve T cells, memory T cells, Treg cells and Th17 cells in HDs and ACS patients. Data are representative of 5 independent experiments. *(B)* Overlay and heatmap summary of STAT3 phosphorylation in immune cell subtypes from PBMCs defined as: myeloid cells, lymphocytes, B cell, CD4^+^ T cells, naïve T cells, memory T cells and Treg cells in ACS patients (n = 10) with different levels of IL-6 and TGF-β1. The color scale indicates the difference in the log2 mean intensity of pSTAT3. *(C)* Statistical analysis of the expression of the pSTAT3 levels in T cell subsets from ACS patients (n = 10) with different levels of IL-6 and TGF-β1 (Figure S3). *(D)* Correlation of individual Th17 and Treg cells with the levels of pSTAT3 in ACS patients (n = 25). The relationships are also depicted using linear regression (solid line) with 95% confidence bands (interrupted lines). *(E)* Averaged *RORγ*t and *FOXP3* mRNA expression levels in T cell subsets from ACS patients (n = 10), as determined by real time PCR from ACS patients, normalized with *GAPDH* mRNA levels. Representative FCM results. *(F)* Inducing Th17 cell from naïve T cells and memory T cells with ACS serum. Cells were purified from HD PBMCs by MACS and co-cultured with selective ACS serum (containing high level IL-6 and TGF-β1), as previously described. Data are representative of 5 independent experiments. *: *P*<0.05 is considered statistically significant.

Given that IL-6 promotes STAT3 phosphorylation following binding to the gp130 receptor [26] and that phosphorylated STAT3 (pSTAT3) directly regulates IL-17 transcription, we sought to characterize the levels of pSTAT3 in the ACS and HD groups. STAT3 was strongly activated in naïve T cells and was less activated in memory T, Treg and Th17 cells from ACS patients, compared with HDs (Fig. 3A). To define the relationship between the levels of pSTAT3 and that of IL-6 and TGF-β1 across immune cell types, we next analyzed pSTAT3 levels in specific immune cell types from PBMCs from 10 ACS patients with different levels of these cytokines using flow cytometry coupled to analyses with Cytobank software (Fig. 3B). The pSTAT3 levels across cell types (with the exception of B-cells), and most particularly for naïve T cells, correlated with IL-6 levels among patients, in that higher IL-6 levels resulted in increased pSTAT3 levels (Fig. 3B and C). In addition, high levels of TGF-β1 were concurrent with lower levels of pSTAT3 levels in ACS patients (Fig. 3C). Moreover, individual pSTAT3 levels in CD4^+^ T cells positively correlated with an increased frequency of Th17 cells in patients with ACS but did not correlate with frequencies of Treg cell populations (Fig. 3D). These results suggest a direct implication of pSTAT3 levels in the enhanced expression of IL-17 in CD4^+^ T cells from ACS patients. The STAT3-regulated RORγt and its protein RORγt is also known to regulate IL-17 transcription [27]. Similar to that of pSTAT3, RORγt mRNA levels were significantly higher in naïve T cells from ACS patients compared with HDs (Fig. 3E upper). In contrast, *FOXP3* mRNA expression was significantly reduced in ACS naïve T cells compared with HDs (Fig. 3E lower). To confirm whether Th17 are derived from naïve T cells under ACS disease conditions, naïve T cells and memory T cells were purified from HD PBMCs by MACS and co-cultured with selective ACS serum (containing high level IL-6 and TGF-β1), as previously described. Th17 cell levels were significantly increased when incubated with ACS serum and naïve T cells rather than memory T cells (Fig. 3F). In addition, induced Th17 cells consisted of a specific population of Foxp3^+^IL-17^+^ double-positive T cells. Overall, naïve T cells from ACS displayed higher pSTAT3 and RORγt expression compared with HDs, and increased pSTAT3 levels correlated with higher Th17 cell frequencies. These results indicate that the increased naïve T cell activation was presumably mediated by the systemic inflammatory state in ACS and specifically by the IL-6/STAT3 signaling pathway.

### IL-6-STAT3 signaling blocking prevents Th17 cell differentiation from naïve CD4^+^ T cells

**Figure 4:**
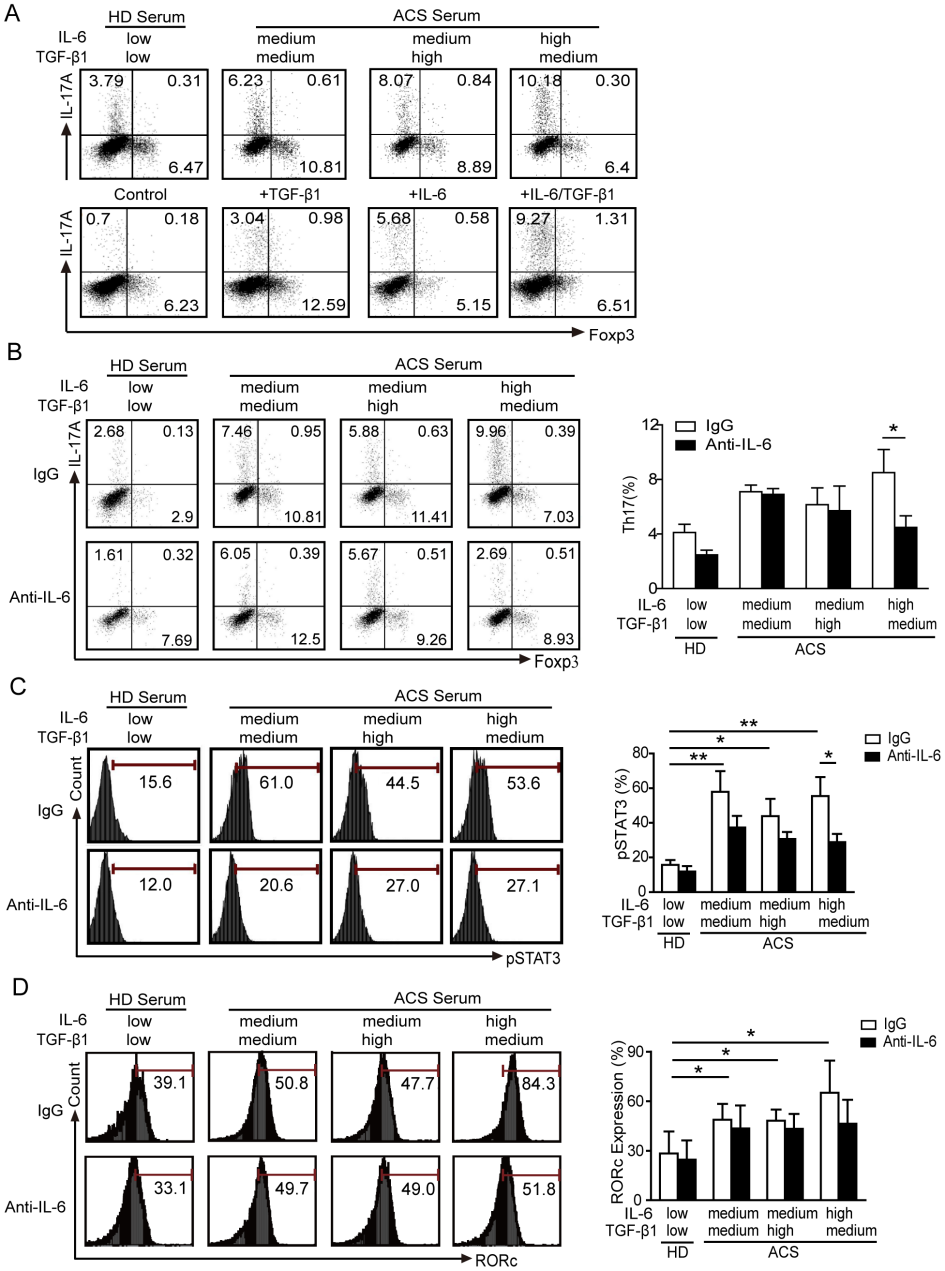
IL-6 neutralization prevents Th17 cell differentiation from naïve CD4^+^ T cells. Representative FCM results. *(A)* Frequencies of Th17/Treg cells detected in PBMCs from HD after stimulation with ACS patient sera (upper panel) and with the indicated cytokines (lower panel). Cells cultured *in vitro* with plate bound anti-CD3 and soluble anti-CD28. Data are representative of 5 independent experiments. *(B-D)* Naïve T cells from HDs were initially obtained by MACS and cells were stimulated in the presence of sera from allogeneic HDs or patients with varying levels of IL-6 and TGF-β1, as previously described. Shown are the IgG control (upper panel) and the anti-IL-6 treatment (lower panel). After 7 days of culture, the frequencies of Th17 and Treg cells *(B)* and the levels of pSTAT3 *(C)* and RORγt *(D)* were measured by FCM. *: *P*<0.05 is considered statistically significant.

To test whether IL-6 and/or TGF-β1 were involved in the differentiation of IL-17-producing T cells, we treated CD4^+^ T cells from the ACS patients with several cytokines, including TNF-α alone or in combination with TGF-β1 (data not shown). However, none of these factors induced the differentiation of IL-17-producing T cells, suggesting the unique role of IL-6 for this effect (data not shown). To further define the action of IL-6 and TGF-β1 on Th17 differentiation, the *ex vivo* induction of Th17 proliferation was analyzed in PBMCs from HDs treated with sera from ACS patients with different levels of IL-6 and TGF-β1 (Fig. 4). Sera with high IL-6 and medium TGF-β1 levels upregulated IL-17 expression to levels comparable to those obtained with stimulation by recombinant hIL-6 and TGF-β1 (Fig. 4A). We next investigated the contribution of IL-6 and TGF-β1 on the differentiation of naïve CD4^+^ T cells into Th17 cells. As shown in Figure 4B, the differentiation of Th17 cells from naïve CD4^+^ T cells was markedly induced by ACS sera with high IL-6 levels. This effect was specifically related to IL-6, as shown by the suppression of Th17 cell differentiation with an anti-IL-6 antibody treatment. Nevertheless, the effect of the anti-IL-6 antibody on Th17 cell differentiation was not visible in cells treated with ACS sera with moderate levels of IL-6 (Fig. 4B). Antibody-mediated depletion of IL-6 strongly reduced the levels of pSTAT3 with all groups of ACS sera (Fig. 4C). Furthermore, IL-6 neutralizing antibody in the presence of serum from ACS patients with high IL-6 levels also blocked the induction of RORγt (Fig. 4D). The elevated frequencies of Th17 cells that were observed in the group with high IL-6 levels, as well as robust the activation of STAT3, suggest that naïve CD4^+^ T cells persistently exposed to IL-6 *in vivo* and *in vitro* retain their ability to differentiate into Th17 cells. Collectively, these data directly implicate IL-6-STAT3 signaling in the regulation of Th17 cell differentiation from naïve T cells during ACS.

## Discussion

**Figure 5:**
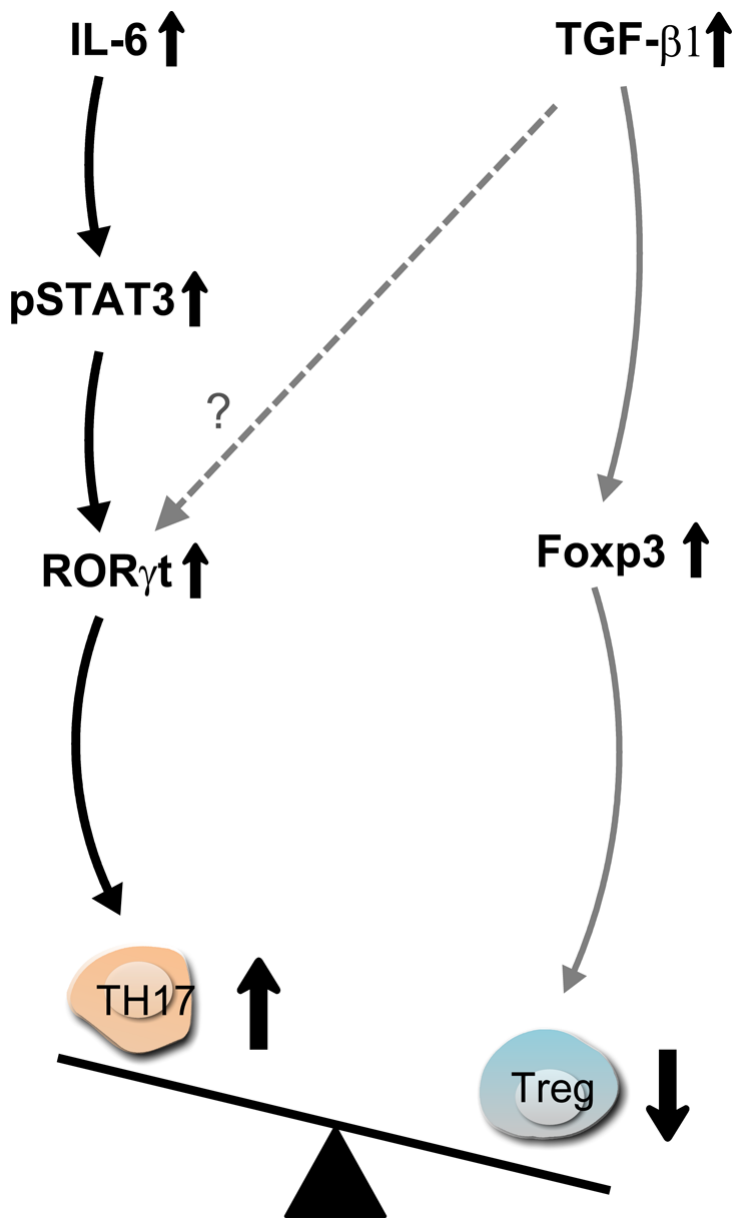
Schematic representation of the pathways regulating the frequencies of Th17/Treg cell populations in ACS. IL-6-activated STAT3 signaling upregulates RORγt to promote Th17 cell differentiation through naïve T cells. High levels of TGF-β1 in ACS patients promote Treg cells differentiation but may inhibit Th17 cell differentiation. Imbalanced Th17 and Treg cell frequencies in ACS patients may originate from the reciprocal modulations by IL-6 and TGF-β1.

Chronic and acute coronary inflammation as potential triggers of ACS provide new insight into mechanisms of disease [28]. Increased circulating IL-17 levels have been previously reported in patients with ACS compared to subjects with stable angina and healthy controls [29−30], but it is not entirely clear how IL-17-producing Th17 cells are generated. This work investigated the mechanism underlying the dysregulation of Th17 and Treg cell differentiation in ACS. We demonstrated that in the inflammatory environment of ACS, elevated levels of IL-6 contributed to the induction of Th17 cell generation from naïve T cells and were negatively correlated with Treg cell levels. Elevated IL-6 levels correlated with increased pSTAT3 and RORγt levels, which are critical for the development of IL-17-producing Th17 cells (Fig. 5).

The initial aim of this study was to determine whether an imbalance existed between Th17 and Treg cell populations as a characteristic of patients suffering from ACS, as was previously suggested ^5^. The inflammatory response is considered to be a pathogenetic component of ACS. Immune cells, including T cells, macrophages, NKT cells, dendritic cells, and mast cells, infiltrate lesions at all stages. The mechanisms of inflammatory effectors depend on largely through cytokine secretion. Anti-inflammatory and immunoregulation mechanisms (such as anti-inflammatory cytokines, protective antibodies and regulatory T cells) may inhibit inflammatory progress and may be attractive targets for disease prevention and/or treatment in ACS. We confirmed that there were increased Th17 and decreased Treg cell population frequencies in patients with ACS. These data suggest that Th17 cells may participate in the inflammatory process during ACS [31]. We hypothesize that such an imbalance between the Th17/Treg cells may be driven by the cytokine microenvironment.

Increased circulating levels of IL-6 in patients with UA compared to patients with stable angina was first reported in 1996 [32]. However, in this study, a high level of IL-6 was found in ACS patients, especially in the STEMI and NSTEMI groups and IL-6 was the cytokine observed to correlate with the increased frequency of Th17 cells. IL-6 is released through the stimulation of other cytokines, such as IL-1β and TNF-α [33]. The raw data showed increased levels of IL-1β and TNF-α compared to the HDs, but there was no significant difference. The time intervals between the release of IL-1β, TNF-α or other cytokines and IL-6 were not investigated in our study, which may explain the absence of correlation between IL-1β, TNF-α and IL-17. IL-6 has a longer half-life than TNF-α and IL-1β and its blood level remains consistently elevated in the presence of various diseases [34−35].

IL-6 is a potent activator of STAT3. Given the regulatory role of STAT3 in IL-17 expression, we examined the role of STAT3 activation in the modulation of Th17 levels in ACS patients. We observed higher levels of STAT3 tyrosine phosphorylation in naïve T cells than in memory T cells from ACS patients. Relying on the neutralizing antibody depletion of IL-6 in ACS sera, we demonstrated that IL-6, through STAT3 activation, contributed to the differentiation of naïve CD4^+^ T cells into Th17 cells in ACS. It is worth noting that we cannot rule out the possibility that cytokines other than IL-6, such as IL-10, IL-27 and IL-21, participate in STAT3 activation in cells from ACS patients [36]. Intriguingly, both pro-inflammatory IL-6 and anti-inflammatory TGF-β1 increased in ACS patients. It is possible that IL-1β and IL-23 levels, combined with TGF-β1, may contribute to the expansion of Th17 cells from naïve T cells [36]. Reports have shown that TGF-β1 also participates *in vitro* in Th17 cell differentiation from naïve CD4^+^ T cells both in humans [10−11], and mice [12], [18], [37]. However, the difference between the potency of TGF-β1 in human and mouse Th17 cell differentiation was notable. Critically, in these earlier studies, the amount of TGF-β1 that was added to the culture and/or present in the serum was much higher than the physiological levels found *in vivo*. Our results indicate that physiologically relevant high levels of TGF-β1 in sera from ACS patients inhibited the effects of IL-6 and reduced Th17 cell differentiation. This implies a critical interplay between these two cytokines in the modulation of Th17 cell differentiation.

There is currently a great deal of interest in defining the pathogenic consequences of IL-17 because of conflicting results in human atherosclerosis studies [38]. Increased Th17 cells in peripheral blood have been observed in patients with ACS [5], and have been reported to be associated with the severity of carotid artery plaques [41]. Recent clinical research has found that lower levels of circulating IL-17 are significantly associated with a higher risk of major cardiovascular events, including all-cause death and recurrent myocardial infarction (MI), after adjustment for other known prognostic factors, including C-reactive protein and statin treatment [42]. However, IL-6 levels were positively associated with a worse outcome [34], [43]. But at the onset of STEMI, patients can present with very high circulating IL-6 levels (IL-6^+^) or very low IL-6 levels (IL-6^−^). Furthermore, IL-6^+^IL-10^+^ and IL-6^−^IL-10^+^ STEMI patients had similar extents of myocardial damage (defined by TnI levels) [43]. Two large genetics consortia analyses provide the first real evidence that the interleukin-6 (IL-6) receptor protein has a causal role in the development of coronary heart disease (CHD) [44]–[45]. These important findings indicate that targeting IL-6-receptor-mediated signaling may be an effective pharmacological intervention in heart disease. Our findings indicated that an effect of IL-6 was observed in subjects with very high levels of IL-6, which were partially blocked by anti-IL-6R neutralizing antibodies, indicating the possibility that other cytokines that induce IL-6 might be of major importance in other ACS patients. The patients with extremely high concentrations of IL-6 were mainly concentrated in the STEMI and NSTEMI groups. Although the median content of IL-6 in the STEMI group was higher than in the NSTEMI group, there was no significant difference between the two groups. In addition to the inflammatory cause accounting for IL-6 increase, the effect of polymorphism in the IL-6 gene [46] or related genes [47] on IL-6 transcription and blood IL-6 levels should be considered and needs further study. In addition to an effect on Th17 cell frequencies, the IL-6 levels in sera from ACS patients negatively correlated with the frequency of Treg cells, which was concurrent with a decreased expression of FOXP3 expression in Treg cells. Conversely, TGF-β1 levels positively correlated with the frequency of Treg cells. Nevertheless, while strongly reducing Th17 cell frequencies, antibody-mediated IL-6 depletion in ACS sera did not increase Treg cell frequencies, suggesting that IL-6 was not essential in contributing to the repression of FOXP3 expression in Treg cells in ACS patients.

In summary, our findings identify a critical role for IL-6/STAT3 signaling in the regulation of the balance of Treg/Th17 cells in ACS. These results establish an essential contribution of the cytokine environment in the generation of Th17 cells. The determination of whether Th17 cells further deteriorate or ameliorate inflammation during the development of ACS requires additional studies. In this study, increased Th17/Treg cell frequencies were observed in ACS patients due to the increased differentiation of Th17 cells from naïve T cells through heightened circulating IL-6 levels. Biologics, including anti-IL-6R agents, are used as drugs to reduce inflammation [48]. IL-6 neutralization strategies can suppress auto-immune arthritis through the inhibition of inflammatory Th17 responses [49] more efficiently than anti-TNF-α therapy [50]. As drugs that target IL-6 have been used in the clinic [51], it will be interesting to determine how these drugs affect the abundance, phenotype, and functional activity of Treg/Th17 cells in immune-mediated disease and whether these drugs present novel therapeutic avenues for the treatment of ACS.

## Supporting Information

File S1 Supporting Information.
(DOC)
