# Supplementary material for: Imbalanced Frequencies of Th17 and Treg Cells in Acute Coronary Syndromes Are Mediated by IL-6-STAT3 Signaling
Source: PLoS One. 2013 Aug 26;8(8):e72804. doi: 10.1371/journal.pone.0072804 (PMC3753235; doi:10.1371/journal.pone.0072804)
Supplement: File S1 — Supporting Information. (DOC) [file pone.0072804.s001.doc]

**Supporting Information**


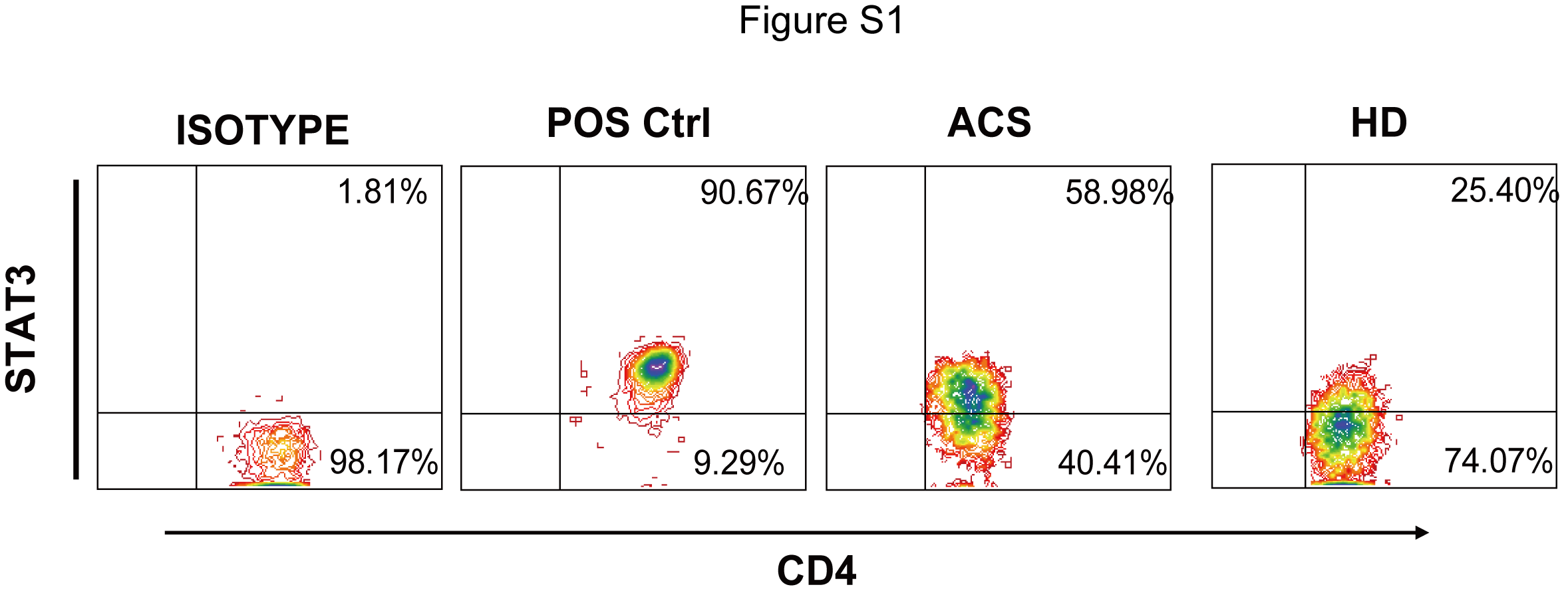


Figure S1. Phospho Flow Cytometry Methods for the Analysis of STAT3 in PBMCs. For analysis the phospholation levels of Stat3 in CD4 T cell, PBMCs were stained with isoptype or were treated with IL-6 (100 ng/mL) as positive cotrol. Cells were fixed by 16% formaldehyde (final concentration of ~1.5%) for 30min, permeabilized by 1 mL of ice-cold methanol for 30 min on ice, and stained with phospho-specific antibodies against Stat3 (pY705).


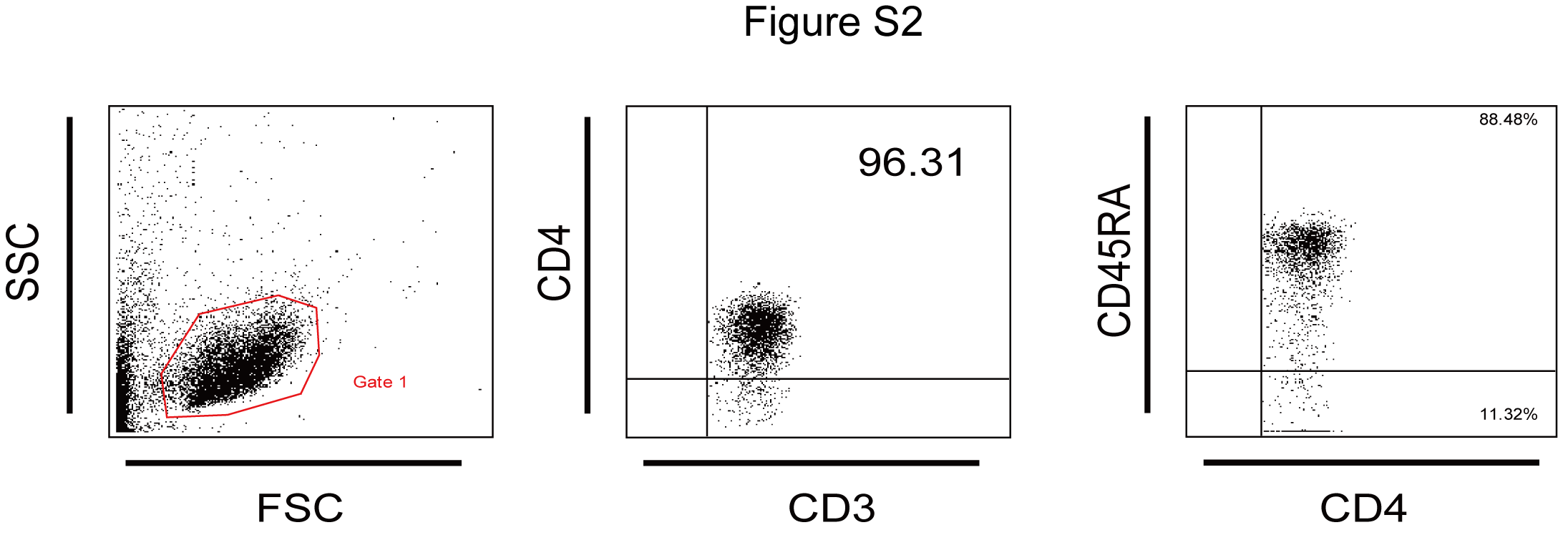


Figure S2. MACS isolation of naive CD4 T cells. A negative-selection procedure developed by Miltenyi and colleagues was chosen for purification of human naive CD4^+^ T cells. The effectiveness of the negative selection was evaluated by three-color FACS analysis using antibodies to CD3, CD4 and CD45RA. Cells were examined by forward scatter (FSC) and side scatter (SSC) to identify the viable cell fraction (left). The percentage of cells expressing CD3^+^CD4^+^T cells (middle), Naive T cell (right) is shown in the appropriate quadrants. Each plot represents a typical analysis from 20 experiments.


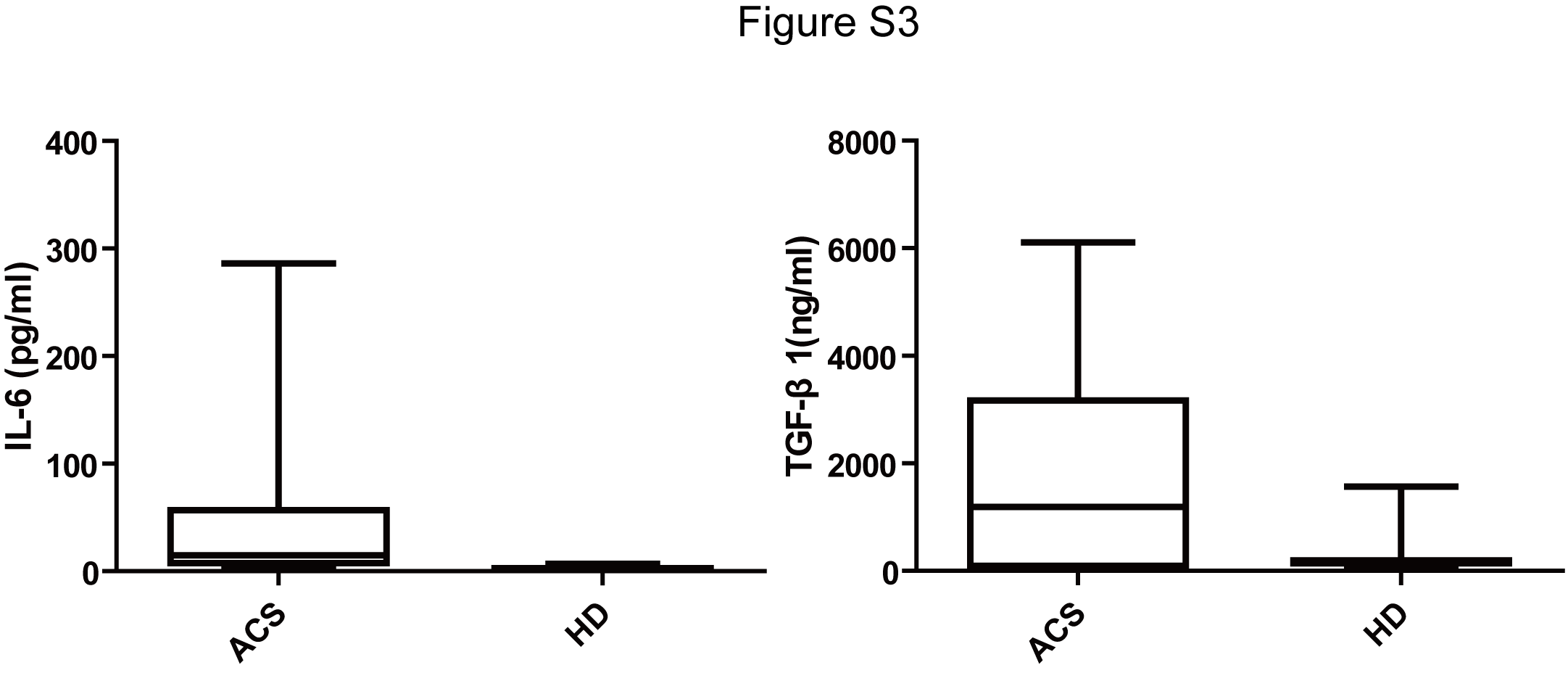


Figure S3. The serum levels of IL-6 and TGF-β1 in patients with ACS and HD. IL-6 and TGF-β1 were detected by ELISA and higher cytokines were found in ACS than that in HD (*, p<0.05). ACS groups were classified according to serum levels of IL-6 and TGF-β1.

Table S1. Summary of the Real-Time Polymerase Chain Reaction (PCR) primers sequences.

| Gene | Forward | Reverse |
| --- | --- | --- |
| IL-17A | AGAGATCCTGGTCCTGCGCA | GTGACACAGGTGCAGCCCAC |
| Foxp3 | CTACGCCACGCTCATCCGCTGG | GTAGGGTTGGAACACCTGCTGGG |
| RORγt | ACCACCCCCTGCTGAGAAGGAC | TGCACCCCTCACAGGTGATAACCC |
| GAPDH | ATTCCACCCATGGCAAATTC | GCATCGCCCCACTTGATT |
